# Functional and morphologic dysfunctions in the airways of rats submitted to an experimental model of obesity-exacerbated asthma

**DOI:** 10.1038/s41598-022-13551-0

**Published:** 2022-06-09

**Authors:** Sarah Rebeca Dantas Ferreira, Rayane Fernandes Pessoa, Indyra Alencar Duarte Figueiredo, João Pedro Moura Lima, Thayna Maria Costa Fernandes de Moura, Cleyton Oliveira Bezerra, Alissa Maria de Oliveira Martins, Leila Moreira de Carvalho, Marta Suely Madruga, Hassler Clementino Cavalcante, Jailane de Souza Aquino, José Luiz de Brito Alves, Adriano Francisco Alves, Luiz Henrique César Vasconcelos, Fabiana de Andrade Cavalcante

**Affiliations:** 1 Instituto de Pesquisa em Fármacos e Medicamentos, Universidade Federal da Paraíba, João Pessoa, PB Brazil; 2 Programa de Pós-Graduação em Produtos Naturais e Sintéticos Bioativos, Centro de Ciências da Saúde, Universidade Federal da Paraíba, João Pessoa, PB Brazil; 3 Departamento de Engenharia de Alimentos, Centro de Tecnologia, Universidade Federal da Paraíba, João Pessoa, PB Brazil; 4 Centro de Ciências da Saúde, Universidade Federal da Paraíba, João Pessoa, PB Brazil; 5 Departamento de Nutrição, Centro de Ciências da Saúde, Universidade Federal da Paraíba, João Pessoa, PB Brazil; 6 Departamento de Fisiologia e Patologia, Centro de Ciências da Saúde, Universidade Federal da Paraíba, Cidade Universitária, João Pessoa, PB Brazil

**Keywords:** Biological techniques, Molecular biology, Diseases, Pathogenesis

## Abstract

The obesity-exacerbated asthma phenotype is characterized by more severe asthma symptoms and glucocorticoid resistance. The aim of this study was to standardize an obesity-exacerbated asthma model by a high glycemic level index (HGLI) diet and ovalbumin (OVA) sensitization and challenges in Wistar rats. Animals were divided into groups: control (Ctrl), obese (Ob), asthmatic (Asth), obese asthmatic (Ob + Asth) and obese asthmatic treated with dexamethasone (Ob + Asth + Dexa), and in vivo and in vitro functional and morphological parameters were measured. After HGLI consumption, there was an increase in body weight, fasting blood glucose, abdominal circumferences, body mass index and adiposity index. Respiratory function showed a reduction in pulmonary tidal volume and ventilation. In isolated tracheas, carbachol showed an increase in contractile efficacy in the Ob, Ob + Asth and Ob + Asth + Dexa, but mostly on Ob + Asth. Histological analysis of lungs showed peribronchovascular inflammation and smooth muscle hypertrophy and extracellular remodeling on Ob + Asth and Ob + Asth + Dexa. An obesity-exacerbated asthma model was successfully established. Therefore, this model allows further molecular investigations and the search for new therapies for the treatment and relief of symptoms of patients with obesity-induced resistant asthma.

## Introduction

Obesity can be defined as abnormal or excessive accumulation of fat potentially harmful to health. The World Health Organization (WHO) regards obesity as a global epidemic and reports that the majority of the population lives in countries where overweight and obesity kill more people than malnutrition^1^.

Obesity increases the risk of several chronic diseases, including asthma, and acts as a modifying factor. Asthma is characterized by airway hyperresponsiveness, varying degrees of airflow obstruction and pulmonary inflammation, which generate recurrent attacks of shortness of breath and wheezing in patients, which vary in severity and frequency among different subjects^2,3^.

Obesity-exacerbated asthma has been proposed as one of many distinct asthma phenotypes^2^. This association have more frequent and more severe asthma symptoms, in addition to a reduced response to various anti-asthma drugs, including resistance to corticosteroids, such as dexamethasone, referred to as an indicator of severe asthma^4^.

Although the correlation between obesity and asthma has already been reported, several aspects are still not fully elucidated about the exacerbation caused, with factors such as changes in respiratory mechanics and inflammation being cited as some of the main responsible for this worsening^5^.

The use of animal models for the study of obesity-exacerbated asthma is highlighted. Induction of obesity in rodents can be achieved by different strategies, but one of the most used is induction through dietary alteration, which is considered polygenic^6^. The induction of obesity using high glycemic index and load diet (HGLI) in the form of pellets was first cited by Luz et al.^7^, characterized by the increase in gut inflammatory components in adult male Wistar rats. Otherwise, to induce asthma, exposure to allergens, especially ovalbumin, has been one of the most commonly used forms^8^, thus presenting itself as a valuable resource.

Thus, the aim of this study was to associate HGLI diet and ovalbumin-induced respiratory inflammation and to standardize a model of the association between obesity and asthma in Wistar rats and to evaluate changes in the parameters of obesity, tracheal responsiveness, respiratory function, and bronchial morphology that mimic the aggravation of tissue and functional parameters of asthma caused by obesity. After that, this model can be used to understand the mechanisms that involve these two diseases, as well as help in the search for new therapeutic alternatives for their treatment.

## Results

### Diet centesimal composition

The centesimal composition of the standard diet and high glycemic level index diet analyzed in this study are shown in Table 1.

**Table 1:** Centesimal composition of the standard diet (Nuvilab) and HGLI. Data are expressed as the mean ± s.e.m. (n = 3). Test t. *p < 0.05 (standard diet vs. HGLI).

Parameters	Standart diet (g/100 g)	HGLI (g/100 g)
Total moisture and solids	7.80 ± 0.12	16.0 ± 0.11*
Ashes	7.7 ± 0.02	3.6 ± 0.05*
Protein	20.5 ± 0.06	12.3 ± 0.06*
Lipid	8.2 ± 0.07	4.1 ± 0.09*
Glucose	2.1 ± 0.003	19.2 ± 0.07*
Maltose	3.7 ± 0.002	1.9 ± 0.03*
Sucrose	–	5.8 ± 0.03*
Fructose	1.8 ± 0.003	–
Mannose	0.8 ± 0.002	17.6 ± 0.008*
Total carbohydrates	6.7 ± 0.003	44.5 ± 0.1*

### Evaluation of experimental obesity

#### Animal weight evolution, food intake and fasting blood glucose

**Figure 1:**
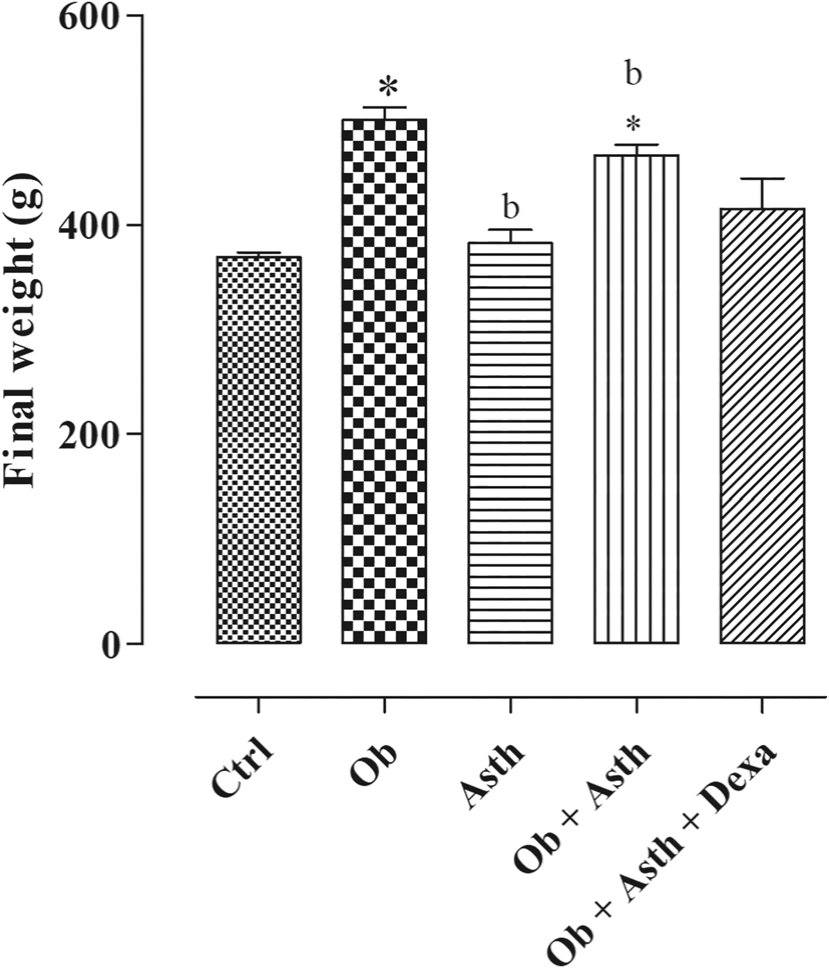
Final body mass values of rats from the Ctrl, Ob, Asth, Ob + Asth and Ob + Asth + Dexa. One-way ANOVA followed by Tukey's posttest (n = 4–6). *p < 0.05 (Ctrl vs. Ob, Ob + Asth), ^b^*p* < 0.05 (Asth vs. Ob + Asth).

The initial body mass of all experimental groups was similar, but after 16 weeks of HGLI consumption, both Ob and Ob + Asth animals had a greater weight than the Ctrl. The Asth, however, did not show any difference compared to the Ctrl but presented lower body mass than the Ob and Ob + Asth. After treatment, Ob + Asth + Dexa did not increase weight when compared to the Ctrl (Table 2, Fig. 1, n = 4–6).

**Table 2:** Initial body mass values, body mass gain and food intake of rats from the Ctrl, Ob, Asth, Ob + Asth and Ob + Asth + Dexa. One-way ANOVA followed by Tukey's posttest (n = 4–6). *p < 0.05 (Ctrl vs. Ob, Ob + Asth), ^b^*p* < 0.05 (Asth vs. Ob + Asth); ^#^*p* < 0.05 (Ob + Asth + Dexa vs. Ob + Asth).

Groups	Initial body weight (g)	Body weight gain (g)	Food consumption (g)
Control	256.0 ± 11.3	113.0 ± 13.1	160.8 ± 3.4
Obese	263.8 ± 15.5	236.2 ± 20.2*	156.6 ± 4.0
Asthma	247.8 ± 12.7	134.3 ± 2.4^b^	167.2 ± 3.4^b^
Obese asthma	268.8 ± 11.8	197.5 ± 17.1*^b#^	150.1 ± 2.9^b^
Obese asthma dexamethasone	317.5 ± 28.4	97.7 ± 19.2	146.9 ± 4.3

The mean weekly body mass gain was also evaluated between the groups over 16 weeks, and it was observed that both the Ob and Ob + Asth groups obtained a greater body mass gain during this period than the Ctrl group. The Asth group showed less weight gain than the groups with dietary changes but did not differ from the control. The group treated with dexamethasone had the lowest weight gain compared to the other groups. Despite these results, the mean weekly food intake was not changed when compared to the control group (Table 2, n = 4–6).

**Figure 2:**
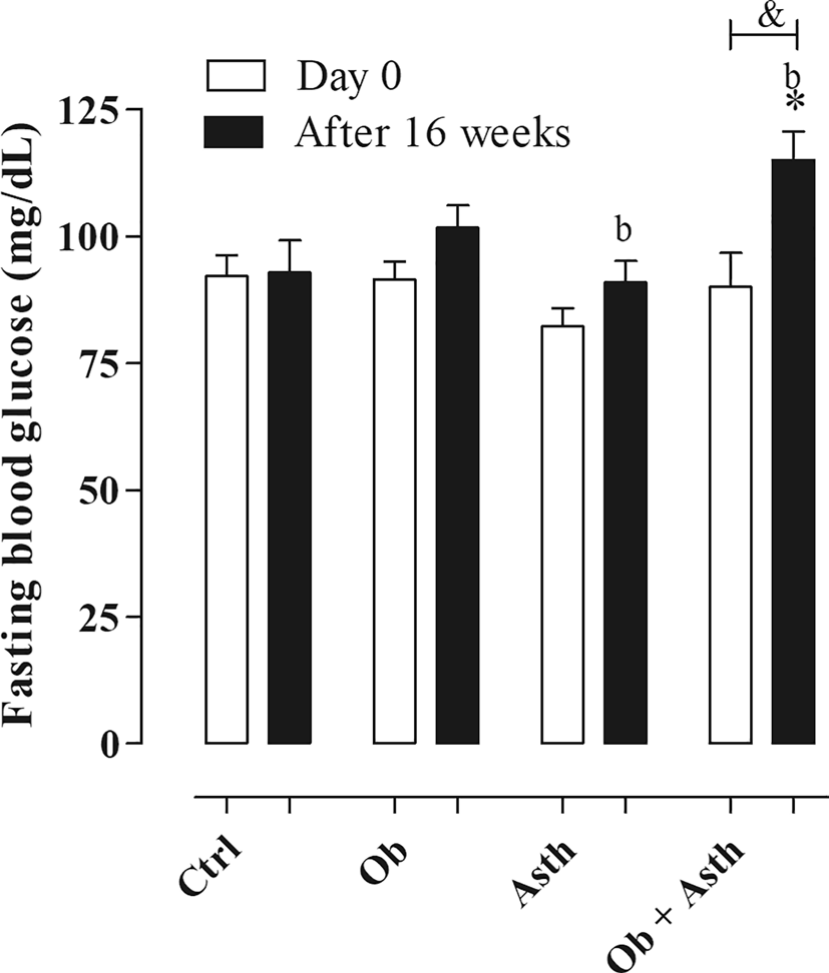
Fasting blood glucose of rats from the Ctrl, Ob, Asth and Ob + Asth. One-way ANOVA followed by Tukey's posttest (n = 6). *p < 0.05 (Ctrl vs. Ob + Asth), ^b^*p* < 0.05 (Asth vs. Ob + Asth), ^&^*p* < 0.05 (Ob + Asth day 0 vs. Ob + Asth after 16 weeks).

Fasting glucose values were similar on experimental groups before disease induction, being 92.1 ± 4.1 mg/dL for the Ctrl, 91.5 ± 3.5 mg/dL for the Ob, 82 0.2 ± 3.7 mg/dL for Asth and 90.0 ± 6.6 mg/dL for Ob + Asth (Fig. 2, n = 6).

After consuming the HGLI diet for 16 weeks, the Ob (101.7 ± 4.4 mg/dL) and Asth (90.8 ± 4.2 mg/dL) groups showed no change in blood glucose levels fasting compared to Ctrl (92.8 ± 6.3 mg/dL). However, the Ob + Asth (115.0 ± 5.6 mg/dL) showed an increase in fasting blood glucose levels when compared to the Ctrl, being higher than the blood glucose of the same animals before the dietary change (Fig. 2, n = 6).

#### Murinometric parameters

##### Lee index, body mass index, chest and abdominal circumferences

**Figure 3:**
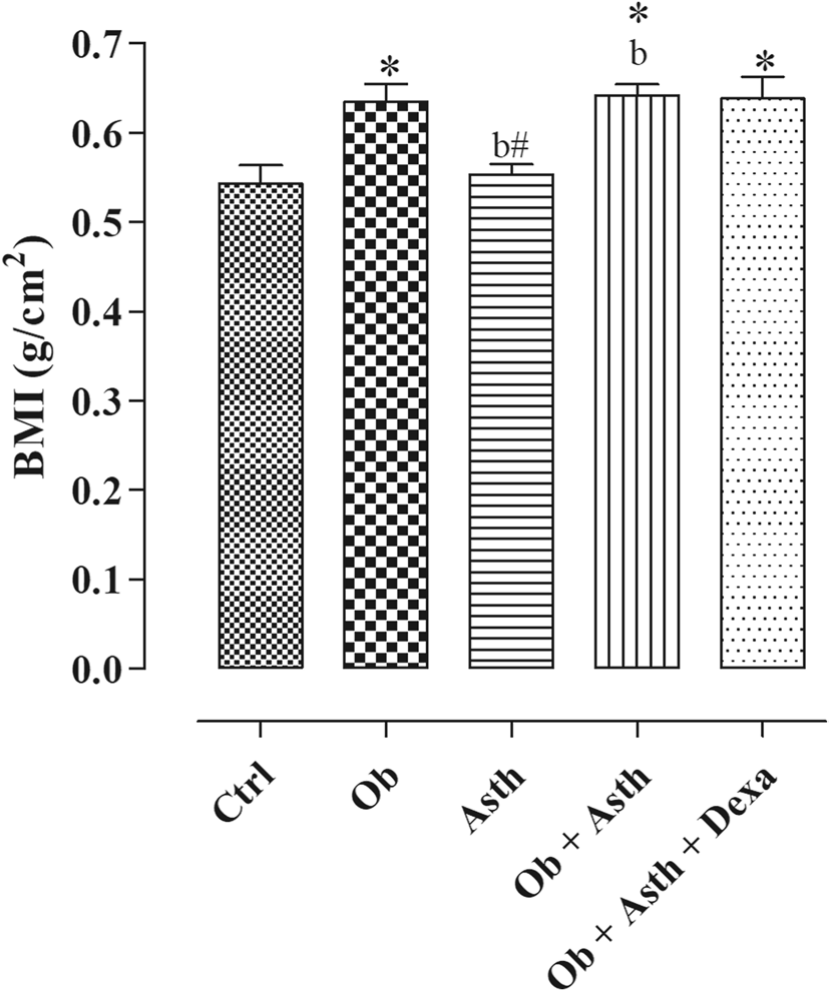
Evaluation of body mass index of rats from Ctrl, Ob, Asth, Ob + Asth and Ob + Asth + Dexa. One-way ANOVA followed by Tukey's posttest (n = 6). *p < 0.05 (Ctrl vs. Ob, Ob + Asth); ^b^*p* < 0.05 (Asth vs. Ob + Asth).

There was no change in the Lee index values between the experimental groups (Table 3, n = 6), but the body mass index was increased in the Ob, Ob + Asth and Ob + Asth + Dexa (Fig. 3, n = 6).

**Table 3:** Values of Lee and body mass index, thoracic and abdominal circumference of rats from Ctrl, Ob, Asth, Ob + Asth and Ob + Asth + Dexa. One-way ANOVA followed by Tukey's posttest (n = 6). *p < 0.05 (Ctrl vs. Ob, Ob + Asth); ^b^*p* < 0.05 (Asth vs. Ob + Asth).

Groups	Lee index (g/cm)	Thoracic circumference (cm)	Abdominal circumference (cm)
Control	0.27 ± 0.004	17.2 ± 0.5	18.8 ± 0.3
Obese	0.28 ± 0.003	18.3 ± 0.8	21.7 ± 0.4*
Asthma	0.27 ± 0.002	17.1 ± 0.3	18.1 ± 0.1^b^
Obese asthma	0.28 ± 0.002	18.0 ± 0.5	21.4 ± 0.1*^b^
Obese asthma dexamethasone	0.29 ± 0.005	16.9 ± 0.3	20.6 ± 0.5*

There was no change in thoracic circumference measurements (Table 3, n = 6); however, the measures of abdominal circumference when compared to the Ctrl were higher in the animals that received HGLI, Ob, Ob + Asth and Ob + Asth + Dexa and did not differ from each other. The Asth, otherwise, did not show any difference when compared to the control; however, they presented lower abdominal circumference when compared to the Ob + Asth and Ob + Asth + Dexa groups (Table 3, n = 6).

##### Mass of adipose tissue deposits and adiposity index

**Figure 4:**
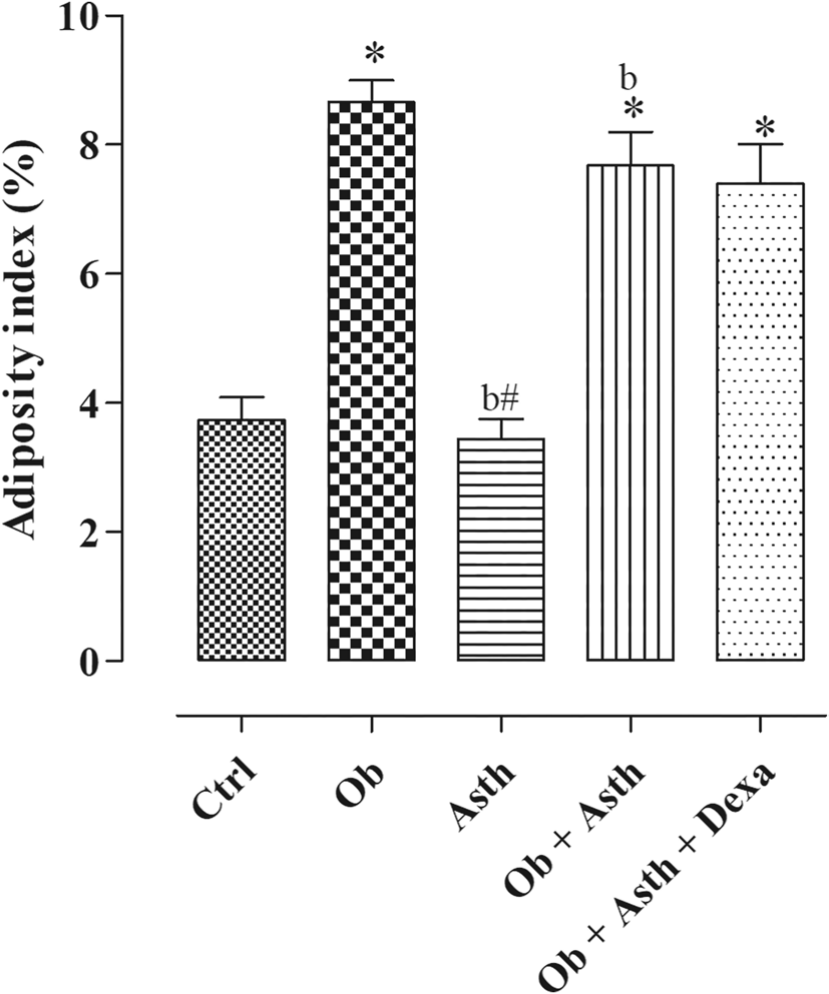
Adiposity index of rats from the Ctrl, Ob, Asth, Ob + Asth and Ob + Asth + Dexa. One-way ANOVA followed by Tukey's posttest (n = 4–6). *p < 0.05 (Ctrl vs. Ob, Ob + Asth, and Ob + Asth + Dexa), ^b^*p* < 0.05 (Asth vs. Ob + Asth).

The inguinal, epididymal, retroperitoneal adipose tissue mass and adiposity index were increased in animals from the Ob, Ob + Asth and Ob + Asth + Dexa compared to the Ctrl and Asth, which showed no difference between them. Furthermore, Asth, in turn, presented lower values for epididymal and retroperitoneal adipose tissue mass and adiposity index than obese and asthmatic animals (Table 4, Fig. 4, n = 6).

**Table 4:** Values of inguinal, epididymal, retroperitoneal adipose tissue mass and adiposity index of rats from the Ctrl, Ob, Asth, Ob + Asth and Ob + Asth + Dexa. One-way ANOVA followed by Tukey's posttest (n = 4–6). *p < 0.05 (Ctrl vs. Ob, Ob + Asth), ^b^*p* < 0.05 (Asth vs. Ob + Asth).

Groups	Inguinal fat (g/100 g)	Epididimal fat (g/100 g)	Retroperitoneal fat (g/100 g)
Control	0.8 ± 0.06	1.5 ± 0.08	1.2 ± 0.1
Obese	2.2 ± 0.3*	3.3 ± 0.3*^a^	3.2 ± 0.3*
Asthma	1.1 ± 0.1	1.0 ± 0.1^b^	1.0 ± 0.1^b^
Obese asthma	2.0 ± 0.3*	2.5 ± 0.1*^ab^	3.4 ± 0.2*^b^
Obese asthma dexamethasone	1.9 ± 0.4*	2.4 ± 0.2*	3.1 ± 0.1*

### Respiratory function analysis

**Figure 5:**
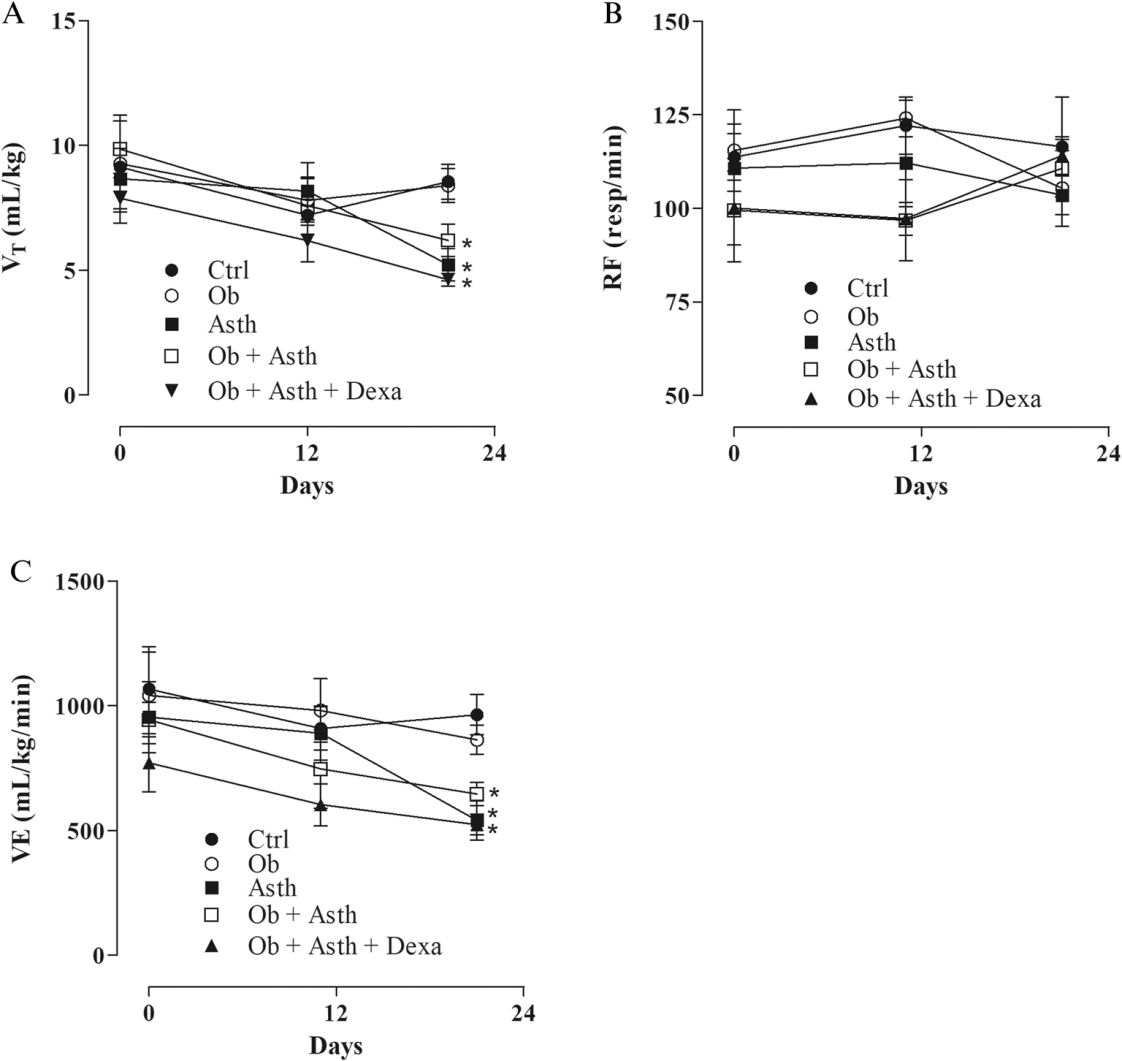
Evaluation of (**A**) tidal volume (VT), (**B**) respiratory frequency (RF), and (**C**) ventilation (VE) on days 1, 12 and 21 of asthma induction. Symbols and vertical bars represent the mean and the e.p.m., respectively. One-way ANOVA followed by Tukey's posttest (n = 6). *p < 0.05 (Ctrl vs. Asth, Ob + Asth and Ob + Asth + Dexa).

Breathing recordings were obtained on days 1, 12 and 21 of asthma induction. Tidal volume was reduced only on the 22nd day on Asth (5.2 ± 0.6 mL/kg), Ob + Asth (6.2 ± 0.6 mL/kg) and Ob + Asth + Dexa (4.6 ± 0.2 mL/kg), showing no difference between them when compared to Ctrl (8.8 ± 0.7 mL/kg) (Fig. 5A). Respiratory frequency was not changed between groups during asthma induction (Fig. 5B). In contrast, ventilation was reduced on the 22nd day in Asth, Ob + Asth and Ob + Asth + Dexa (539.9 ± 78.8; 646.0 ± 47.0; 523.6 ± 42.0 mL/kg/min) compared to Ctrl (964.3 ± 80.5 mL/kg/min), with no difference from OG (863.8 ± 58.6 mL/kg/min) (Fig. 5C) (Supplementary Fig. S1).

### Evaluation of contractile reactivity to ovalbumin in rat trachea—Schultz–Dale reaction

**Figure 6:**
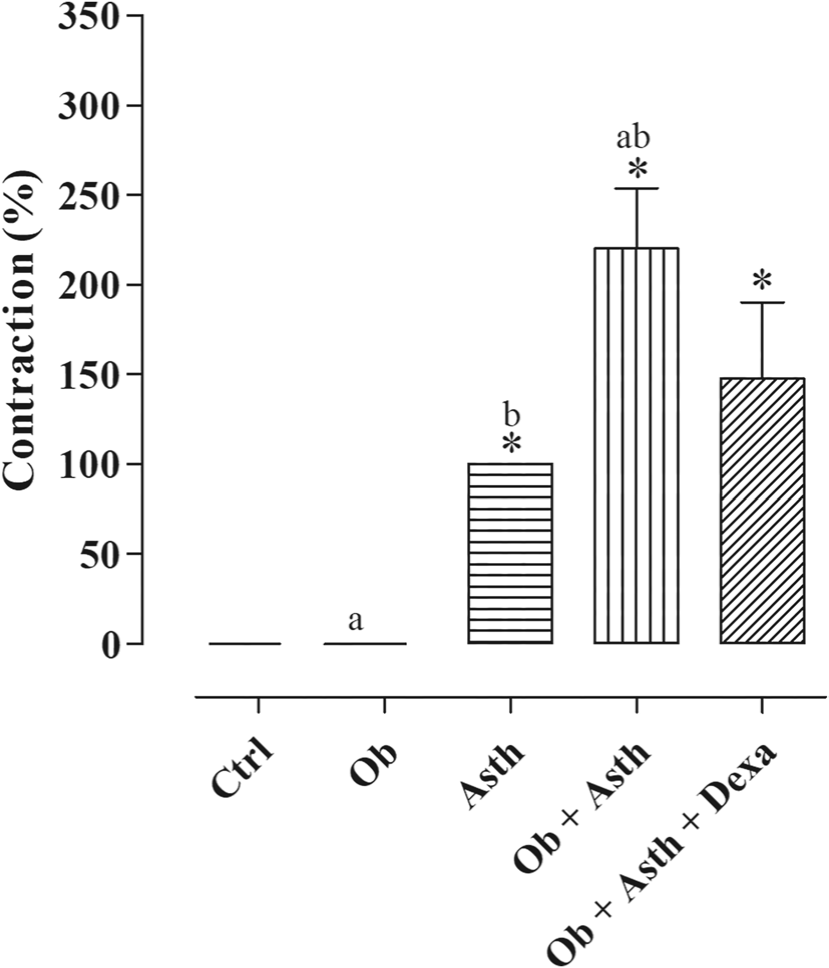
Effect of stimulation of rat trachea with 100 µg/ml OVA. Symbols and vertical bars represent the mean and the e.p.m., respectively. One-way ANOVA followed by Tukey's posttest (n = 3–5). *p < 0.05 (Ctrl vs. Asth, Ob + Asth, and Ob + Asth + Dexa); ^a^*p* < 0,05 (Ob vs. Ob + Asth); ^b^*p* < 0,05 (Asth vs. Ob + Asth).

The rat tracheas of the Ctrl and Ob did not show contractile reactivity to OVA stimulation (E_max_ = 0%) in contrast, in the Asth, Ob + Asth and Ob + Asth + Dexa, OVA promoted contractile reactivity (E_max_ = 100%; 220.0 ± 33.7 and 147.6 ± 42.3%, respectively), where the Ob + Asth showed greater contractile reactivity than the Asth and Ob + Asth + Dexa, which caused a partial reduction in this increase (Fig. 6) (Supplementary Fig. S2).

### Assessment of tracheal contractile reactivity to KCl or CCh in rat trachea

**Figure 7:**
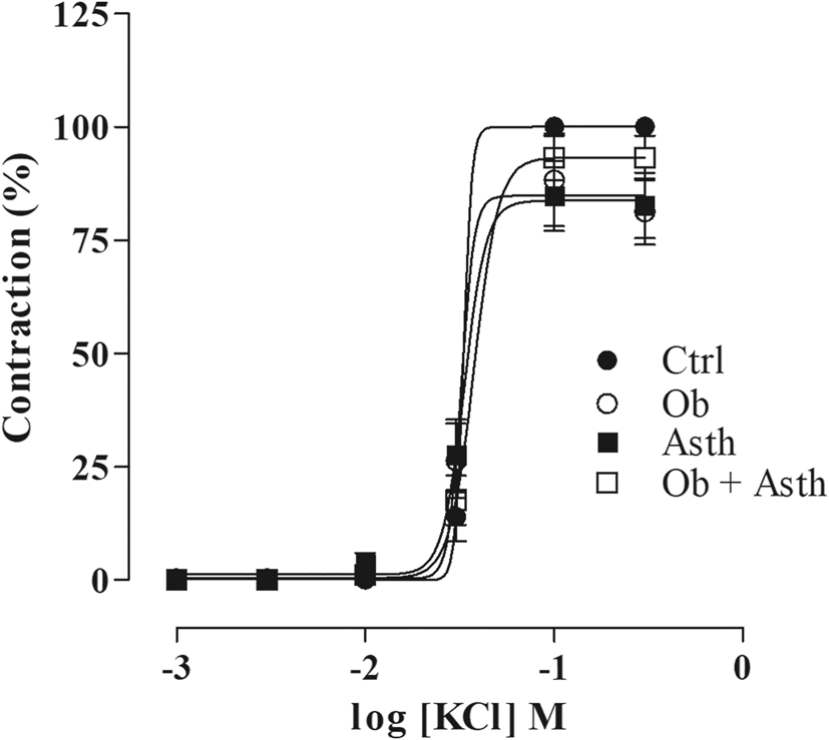
Cumulative concentration–response curves to potassium chloride (KCl) in the presence of functional epithelium in rat trachea. Symbols and vertical bars represent the mean and the e.p.m., respectively. One-way ANOVA followed by Tukey's posttest (n = 5).

The cumulative concentration–response curves for KCl (10^–3^–3 × 10^–1^ M) did not show any change in contractile efficacy and potency between the experimental groups (Fig. 7, Table 5, n = 5) (Supplementary Fig. S3).

**Table 5:** E_max_ and EC_50_ values of contractile and relaxant agents in the Ctrl, Ob, Asth, Ob + Asth and Ob + Asth + Dexa isolated rat trachea groups. One-way ANOVA followed by Tukey's posttest (n = 4–6). *p < 0.05 (Ctrl vs. Ob, Ob + Asth).

Contractile/relaxant agent	Parameter	Control	Obese	Asthma	Obese asthma	Obese asthma dexamethasone
Potassium chloride (KCl)	E_max_ (%)	100.0	88.2 ± 10.2	84.7 ± 7.7	93.1 ± 4.9	–
	EC_50_ (M)	3.3 ± 0.08 × 10^–2^	3.3 ± 0.2 × 10^–2^	3.1 ± 0.3 × 10^–2^	3.5 ± 0.2 × 10^–2^	–
Carbachol (CCh)	E_max_ (%)	100	133.2 ± 10.4*	126.1 ± 2.4*	152.6 ± 5.5*	162.2 ± 7.4*
	EC_50_ (M)	9.7 ± 0.8 × 10^–7^	1.4 ± 0.4 × 10^–6^	1.3 ± 0.5 × 10^–6^	2.6 ± 1.2 × 10^–6^	7.5 ± 1.7 × 10^–7^
Nifedipine	E_max_ (%)	98.5 ± 2.1	96.2 ± 2.4	100.4 ± 3.6	102.2 ± 3.0	–
	EC_50_ (M)	2.2 ± 0.4 × 10^–6^	8.4 ± 3.5 × 10^–6^	7.7 ± 4.9 × 10^–6^	3.5 ± 2.1 × 10^–6^	–
Isoprenaline	E_max_ (%)	76.7 ± 2.6	38.4 ± 2.8*	30.5 ± 2.4*	41.1 ± 5.0*	–
	EC_50_ (M)	1.1 ± 0.4 × 10^–4^	1.9 ± 0.5 × 10^–5^*	3.6 ± 2.2 × 10^–5^	1.2 ± 0.7 × 10^–5^*	–
Aminophylline	E_max_ (%)	98.8 ± 1.1	91.6 ± 1.6	92.6 ± 2.7	96.9 ± 1.2	–
	EC_50_ (M)	5.7 ± 1.1 × 10^–4^	8.4 ± 0.4 × 10^–4^	7.6 ± 1.1 × 10^–4^	6.4 ± 0.5 × 10^–4^	–

**Figure 8:**
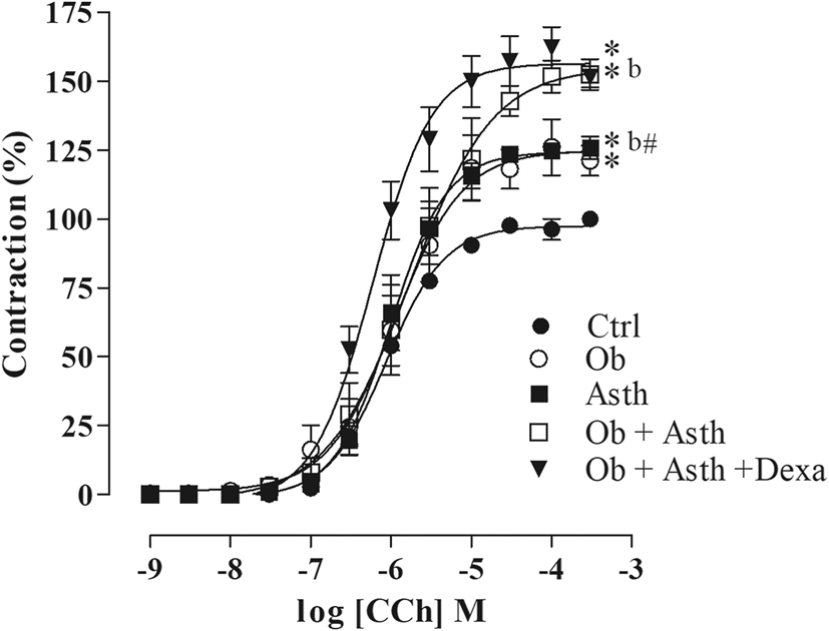
Cumulative concentration–response curves to carbachol (CCh) in the presence of functional epithelium in rat trachea. Symbols and vertical bars represent the mean and the e.p.m., respectively. One-way ANOVA followed by Tukey's posttest (n = 5). *p < 0.05 (Ctrl vs. Asth, Ob, Ob + Asth and Ob + Asth + Dexa); ^b^*p* < 0,05 (Asth vs. Ob + Asth).

The cumulative concentration–response curves for CCh (10^–9^–3 × 10^–4^ M) did not show any change in contractile potency between the groups. However, there was an increase in contractile efficacy in the Ob, Asth, Ob + Asth and Ob + Asth + Dexa compared to the Ctrl, and the animals that presented the two associated diseases (Ob + Asth and Ob + Asth + Dexa) also showed increased efficacy compared to the Asth group (Fig. 8, Table 5, n = 5) (Supplementary Fig. S4).

### Assessment of tracheal relaxant reactivity to nifedipine, isoprenaline or aminophylline in rat trachea precontracted with CCh

**Figure 9:**
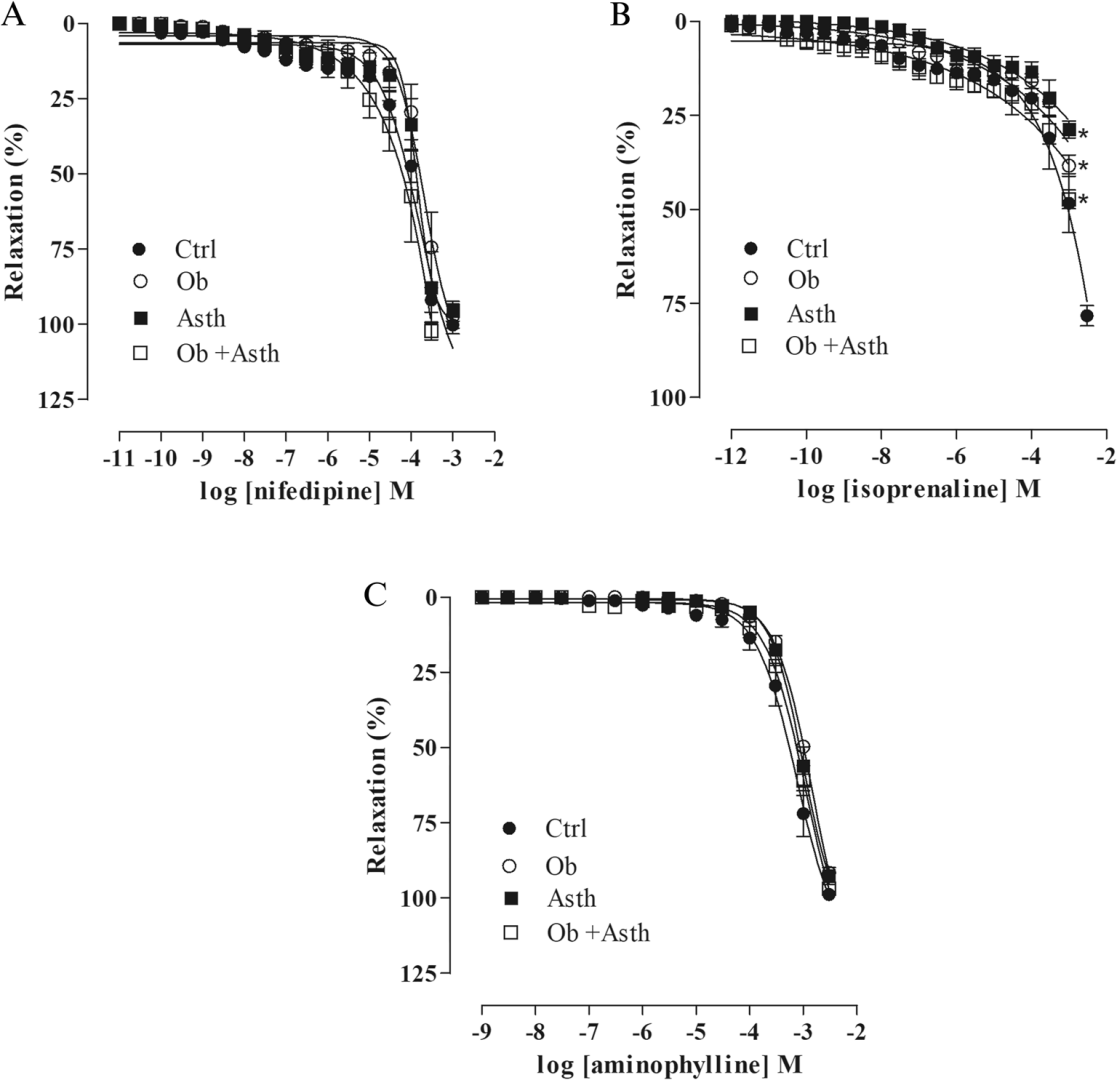
Cumulative concentration–response curves to nifedipine (**A**), isoprenaline (**B**) and aminophylline (**C**) in precontracted rat trachea with 10^–5^ M CCh in the presence of functional epithelium. The symbols and vertical bars represent the mean and the e.p.m., respectively. One-way ANOVA followed by Tukey's posttest (n = 5). *p < 0.05 (Ctrl vs. Ob, Asth and Ob + Asth).

The relaxant tracheal reactivity to nifedipine was equipotent between groups, and the efficacy was also unchanged (Fig. 9A, Table 5, n = 5) (Supplementary Fig. S5).

In contrast, the relaxant tracheal reactivity to isoprenaline showed a reduction in relaxing efficacy in the Ob, Asth and Ob + Asth when compared to the Ctrl. For potency, Ob and Ob + Asth shifted the curve to the right with a reduction in relaxing potency approximately 5.8 and 9.2 times, respectively, when compared to Ctrl, which was equipotent to Asth (Fig. 9B, Table 5, n = 5) (Supplementary Fig. S6).

The relaxant tracheal reactivity to aminophylline was equipotent between groups, and the efficacy was also unchanged (Fig. 9C, Table 5, n = 5) (Supplementary Fig. S7).

### Morphologic evaluations

#### Evaluation of the effects of obesity and asthma on pulmonary

**Figure 10:**
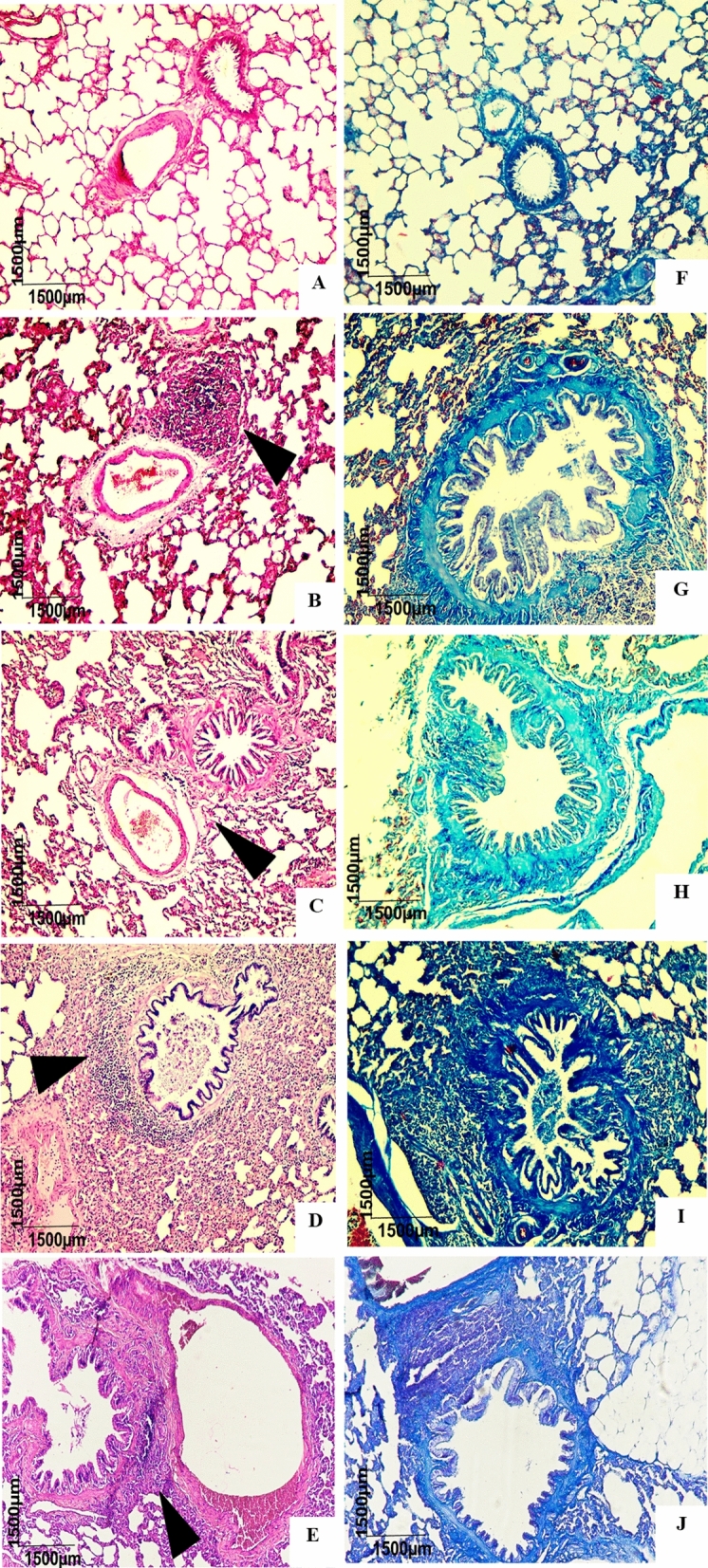
Photomicrograph of the lungs of rats from the Ctrl (**A**,**F**), Ob (**B**,**G**), Asth (**C**,**H**), Ob + Asth (**D**,**I**) and Ob + Asth + Dexa (**E**,**J**) stained with HE and Masson's trichrome, respectively. Cell infiltrate (black arrows).

**Figure 11:**
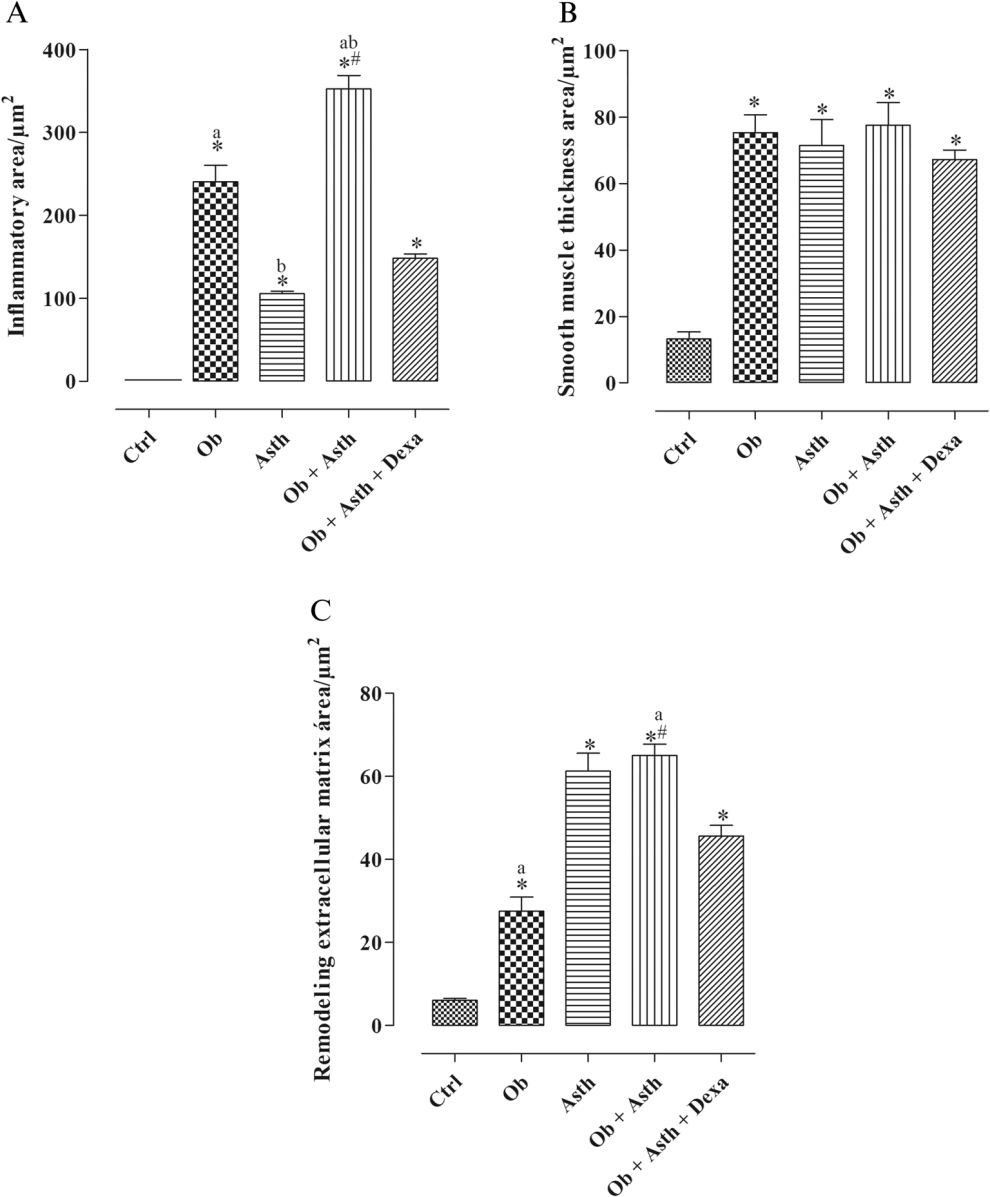
Measurements of inflammatory area (**A**), smooth muscle thickness (**B**) and remodeling through the extracellular matrix (**C**) in the lungs of animals in the Ctrl, Ob, Asth, Ob + Asth and Ob + Asth + Dexa. Symbols and vertical bars represent the mean and the e.p.m., respectively (n = 6). One-way ANOVA followed by Tukey's posttest. *p < 0.05 (Ctrl vs. Ob, Asth and Ob + Asth), ^a^*p* < 0.05 (Ob vs. Ob + Asth), ^b^ *p* < 0.05 (Asth vs. Ob + Asth), ^#^*p* < 0.05 (Ob + Asth vs. Ob + Asth + Dexa).

Histological sections of the lung stained with hematoxylin–eosin (n = 6) showed a parenchyma full of alveolar sacs and structures of the respiratory tree, namely, terminal bronchioles, which have a low columnar epithelium, ciliated with few goblet cells, in addition to pulmonary vessels (Fig. 11A). In the Ob (Fig. 10B), Asth (Fig. 10C), Ob + Asth (Fig. 10D) and Ob + Asth + Dexa (Fig. 10E), it is possible to observe the presence of a mixed and diffuse exudate in the peribronchovascular location, composed mostly of macrophages and eosinophils; however, the animals of the Ob + Asth (Fig. 10D) present a more intense infiltrate with greater destruction of the pulmonary parenchyma than the other experimental conditions.

When Masson's trichrome staining was employed, it was possible to observe the maintenance of the organ stroma with an apparent increase in the extracellular matrix (Fig. 10G–I), while treatment with dexamethasone (Fig. 10J) did not reverse the changes caused by the induction of asthma exacerbated by obesity when compared to the control group (Fig. 10F).

The area of peribronchial inflammation was also evaluated, and it was observed that the Ctrl did not present this type of inflammation, while there was an increase in this parameter in the Ob (240.7 ± 19.5 area/μ^2^) and in the Asth (105.5 ± 3.1 area/μ^2^) and in the Ob + Asth (352.5 ± 16.1 area/μ^2^) compared to the Ctrl, with a decrease after treatment with dexamethasone, but not reversal (148.5 ± 5.2 area/μ^2^) when compared to the Ob + Asth (Fig. 11A, n = 6).

**Figure 12:**
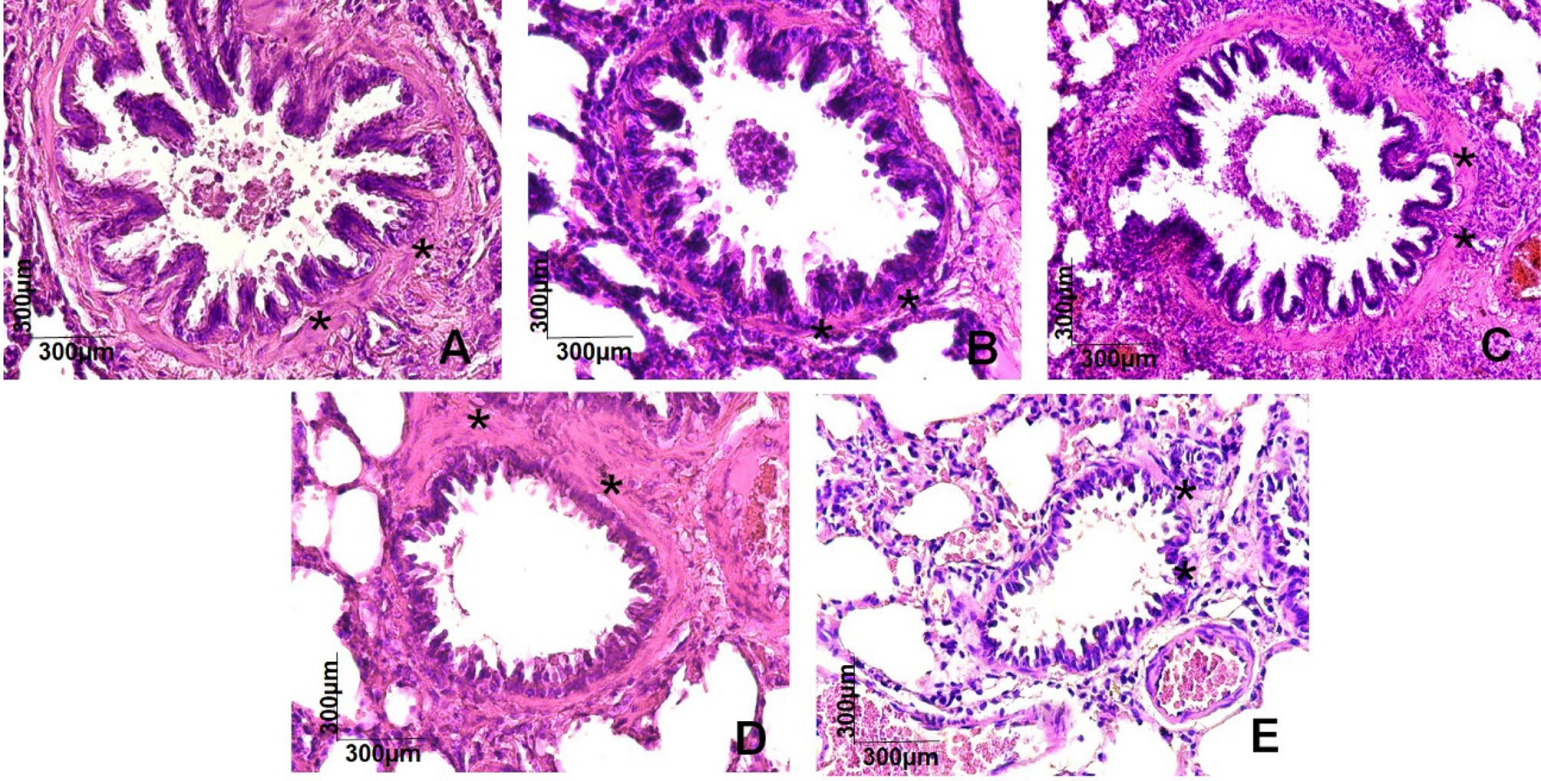
Photomicrograph of smooth muscle of terminal bronchioles of rats from the Ctrl (**A**), Ob (**B**), Asth (**C**), Ob + Asth (**D**) and Ob + Asth + Dexa (**E**) stained with HE. Smooth muscle (asterisk).

The smooth muscle area was also quantified in the experimental groups (n = 6), and it was observed that both the Ob (75.3 ± 5.3 area/μ^2^), Asth (71.5 ± 7.7 area/μ^2^) and Ob + Asth (77.5 ± 6.8 area/μ^2^) showed an increase in this muscle layer, which was not altered by treatment with dexamethasone (67.2 ± 3.9 area/μ^2^) when compared to the Ctrl (13.2 ± 2.1 area/μ^2^) and did not differ from each other (Figs. 11B and 12, n = 6).

Assessing the remodeling area filled by the extracellular matrix (n = 6), when compared to the Ctrl (6.0 ± 0.5 area/μ^2^), there was an increase in Ob (27.5 ± 3.4 area/μ^2^), Asth (61.3 ± 4.2 area/μ^2^) and Ob + Asth (65.0 ± 2.7 area/μ^2^), which showed a reduction of only approximately 30% after treatment with dexamethasone (45.7 ± 2.5 area/μ^2^). Asth and Ob + Asth did not differ from each other and induced greater remodeling when compared to obesity alone (Fig. 11C, n = 6).

## Discussion

Obesity and asthma affect a large portion of the world population and are characterized by increasing predisposition and severity of asthma, making treatment difficult^9^. To understand the main changes caused by this association, in the present work, a standardized model of obesity-exacerbated asthma induced by a high glycemic index diet (HGLI) and ovalbumin was established in Wistar rats.

Individuals who are concurrently affected by both diseases have a clinical and immunological profile that is distinct from other asthma phenotypes^10^. In isolation, asthma has an inflammatory profile characterized by eosinophilic infiltration and high levels of IgE^11^. Obesity-exacerbated asthma usually has a "low-Th2" response, with neutrophilic inflammation and resistance to corticosteroid treatment^12^.

Although the correlation between obesity and asthma has already been reported, several aspects about the exacerbation of asthma when it is associated with obesity, since they are multifactorial diseases, are still not fully elucidated. Changes in respiratory mechanics are mentioned, since the increase in abdominal and thoracic adipose tissue makes it difficult to inflate and comply with the lungs, chest wall and the entire respiratory system, as well as an increase in the production of pro-inflammatory cytokines produced by adipose tissue and increase in reactive oxygen species as some of the main factors responsible for this worsening^5,13–15^.

Thus, characterizing an animal model that easily and reproductibility mimics the changes induced by obesity-exacerbated asthma is important to better understand the pathophysiology of this association allowing research in the field of more effective treatment. For experimental obesity induction, dietary change is a process resembling current human obesity^16^; thus, the HGLI diet chosen for the study presents condensed milk as one of its main components and was offered to the animals for 16 weeks. Masi et al.^17^ reported in a comparative study between a diet rich in fat and the same diet with separate bowl of sweetened condensed milk ad libitum, showed a greater increase in body weight gain in mice, glucose intolerance, increased adipocyte size and cholesterol levels, in addition to being more pro-inflammatory than the diet without the addition of condensed milk, indicating that this ingredient is more inflammatory than fat. Corroborating these data, Luz et al.^7^ used HGLI for the first time and observed increased inflammation in Wistar rats.

The HGLI diet has lower protein and fat content when compared to the standard diet but presents high levels of carbohydrates (Table 1). This type of diet is characterized by a high glycemic index and load, with an increase in blood glucose levels after consumption. High-carbohydrate diets have already been cited produces postprandial hyperinsulinemia, promotes deposition of calories in adipose cells instead of oxidation in lean tissues, and thereby predisposes to weight gain through increased hunger, slowing metabolic rate, or both^18,19^.

In addition, it has already been mentioned that high-protein or high-fat diets produce a greater postprandial satiety hormonal response than diets rich in carbohydrates, which is therefore suitable for inducing obesity^20^.

Thus, among the advantages of a new obese asthma rat model highlighting the easy for induction, the employment of a rich in carbohydrate diet which induces dysfunctions as insulin resistance, hyperglycemia, visceral fat deposition and proinflammatory profile that potentiates the asthma typical inflammation and bronchial remodeling^7,18,19^.

In the present study, after 16 weeks, the Ob and Ob + Asth had a greater final weight and weight gain than the animals that received the control diet (Fig. 1) and were therefore an important factor in the development of obesity. The induction of asthma without changing the diet (Asth) did not cause changes in the weight or body mass of these animals when compared to the Ctrl (Table 2, Fig. 1), indicating that asthma alone does not affect the weight of these animals. These data are similar to those observed in other studies^21–23^.

One of the main managements of asthma is through treatment with corticosteroids, which are steroid hormones that exert potent anti-inflammatory activity^24^; however, it has already been observed that obese asthmatics are less likely to achieve asthma control using corticosteroids^25^. Based on this premise, the animals of the Ob + Asth group were treated with dexamethasone, not to correlate the treatment of asthma, but as a way of proving the implantation of the association of the two diseases.

Thus, the treatment of obese asthmatic animals with dexamethasone (Ob + Asth + Dexa) promoted no difference in weight from the standard diet groups at the end of 16 weeks (Fig. 2); however, there was a marked weight loss during the 5 days of corticosteroid administration, a fact also observed during the induction of asthma (data not shown), indicating that the successive intraperitoneal administrations might cause a temporary loss of appetite, which may be responsible for the weight loss, similar to that observed by Liu et al*.*^26^ in a 20 day continuous intraperitoneal infusion of hydrocortisone sodium succinate in obese and nonobese rats. Despite the mentioned reduction in weight gain, the final weight of these animals was not reduced, indicating that treatment with dexamethasone has little or no effect on obesity parameters.

Changes in HGLI consumption were not observed between the experimental groups (Table 2), indicating that the increase in body weight and mass gain observed occurred due to the increase in the amount of energy ingested and not by the amount of dietary consumption.

The literature already reports that different diets based on their composition may cause controversial changes in the glucose metabolism of the animals that are consuming them, however, it is very common, especially the association of high-calorie diets with hyperglycemia^27^. To assess whether the HGLI diet would cause an increase in glucose levels, this parameter was evaluated. An increase was observed only in the Ob + Asth animals, indicating that somehow, the association of the two diseases can alter glucose metabolism, worsening the clinical condition.

Lee index and BMI were evaluated, and no change in LEE index was observed between groups (Table 3), while there was an increase in BMI in animals that consumed the HGLI diet (Ob, Ob + Asth and Ob + Asth + Dexa) (Fig. 3), similar to demonstrated by Novelli et al*.*^28^, in a high carbohydrate diet offered for rats.

Since these parameters do not discriminate body composition, thoracic and abdominal circumferences were evaluated. Chest circumference was not altered by the change in diet or by the induction of asthma; however, abdominal circumference, in turn, was higher in the Ob, Ob + Asth and Ob + Asth + Dexa, with no difference between them, indicating that asthma does not hinder the increase in circumference caused by HGLI, possibly due to an increase in fat deposition in adipose tissue (Table 3).

Determining the weight of the main fat deposits (inguinal, epididymal and retroperitoneal), as well as the adiposity index, can be a measure of great value for the assessment of obesity. Thus, it was observed that the Ob, Ob + Asth and Ob + Asth + Dexa animals showed an increase in the three fat deposits when compared to the control, leading us to infer that HGLI increased adipose tissue and it can probably configured as a risk factor for worsening asthma. In addition, despite the weight loss caused by treatment with dexamethasone, the main fat deposits were not altered and were resistant to treatment with corticosteroids (Table 4).

The adiposity index was also increased in the Ob, Ob + Asth and Ob + Asth + Dexa compared to the Ctrl and Asth, which did not differ from each other (Fig. 4). Contrary results by Bortolin et al.^29^, who compared some types of obesogenic diets, demonstrated that only the westernized diet would cause an increase in the adiposity rate of these animals.

In the present study, lung function was evaluated, as indicated by the tidal volume, respiratory frequency and ventilation, and it was observed that although the respiratory rate was not altered, there was a reduction in tidal and ventilation (Fig. 5), indicative of reduced airflow characteristic of increased airway resistance, which implies bronchoconstriction promoted by asthma induction.

In a study by Arora et al*.*^30^, a reduction in TV and ventilation was also observed in a model of asthmatic rats; however, in contrast, there was an increase in respiratory rate. Additionally, the tidal volume decreases were related in other models to lung fibrosis and extensive inflammation^31^, which may explain some observed changes. In contrast, rats fed a hypercaloric diet demonstrated an exacerbated increase in respiratory frequency and a decreased expiratory time^32^.

An easy method to functionally confirm the induction of asthma is through a local anaphylactic reaction, based on the premise that in antigenically sensitized animals, when re-exposure to this antigen causes smooth muscle constriction and subsequent increase in airway resistance because of release of contractile mediators, mainly histamine, prostaglandins and leukotrienes^33^.

In functional in vitro measurement, animals previously sensitized (Asth, Ob + Asth and Ob + Asth + Dexa) showed a tracheal contractile response to OVA, whereas the animals of the Ctrl and Ob did not respond to the same stimulus (Fig. 6), confirming functionally asthma implantation in these animals. It is important to point out that Ob + Asth showed a greater contractile effect to OVA when compared to Asth, leading us to suggest an exacerbation of diseases, and the treatment with dexamethasone showed no difference in the Ob + Asth. These data also corroborate previous data related to respiratory function, which showed a reduction in pulmonary ventilation.

Tracheal contractile reactivity assessment using a mainly electromechanical agent potassium chloride (KCl) did not change either efficacy or potency between the experimental groups (Table 5, Fig. 7), indicating that the diseases probably do not present a majority participation of the electromechanical component in hyperreactivity, similar to demonstrated by Vasconcelos et al.^34^ in guinea pig trachea with chronic allergic pulmonary inflammation. In addition, the investigation of the effects of treatment with dexamethasone was carried out only in the pathways that were altered by the induction of obesity-exacerbated asthma.

Knowing that one of the main contractile mechanisms of the airways is promoted by acetylcholine (ACh) via cholinergic innervation^35^, an increase in contractile efficacy was observed in all experimental groups compared with the Ctrl. However, Ob and Asth did not differ from each other, but Ob + Asth showed an increase in contractile efficacy when compared to Asth as well as Ob + Asth (Fig. 8), indicating that both obesity and asthma alone cause tracheal hyperresponsiveness and that the association of the two diseases causes exacerbation and resistance to treatment with dexamethasone.

The increase in contractile reactivity of the trachea in groups of asthmatic rats, being even greater on simultaneously obese and asthmatic animals, has already been observed in other studies using another muscarinic agonist, methacholine Aslani^36^.

To evaluate whether asthma, obesity and the association between the two disorders would alter the electromechanical relaxation pathway, the relaxing effect of the cumulative concentration–response curves to nifedipine, a Ca_V_ channel blocker^37^, was evaluated over the tonic contraction induced by CCh. After that, it was observed that there was no change in efficacy or potency between the groups (Fig. 9A, Table 5), indicating that the diseases do not change the electromechanical component of relaxation.

The pharmacomechanical component of relaxation was evaluated by employing isoprenaline, a β_2_-adrenergic agonist^38^. A reduction in the relaxing efficacy in the Ob, Asth and Ob + Asth was observed; however, the Ctrl did not induce 100% relaxation (Fig. 9B). This event can be explained because despite the expression of β_2_-adrenergic receptors in the airways, they occur mostly in the lower airways, such as bronchioles, when compared to the upper airways, such as the trachea^39^.

Another relaxing mechanism can be evaluated by obtaining a relaxation curve to aminophylline, a nonselective PDE inhibitor^40^, and there was no change in the relaxing efficacy or potency between the groups (Fig. 9C). Altogether, these data indicate that the main changes induced by obesity, asthma and obesity-exacerbated asthma are not due to changes in the tracheal relaxant mechanism.

Through histological analyses of lungs, there was also an increase in the thickness of the smooth muscle layer in all groups, an increase in peribronchovascular inflammation in the asthma group and in even greater levels in obese asthma animals when compared to the Ctrl, as well as greater tissue remodelling by the extracellular matrix in these groups, characterizing asthma and demonstrating that the association of obesity and asthma worsens the inflammatory condition. The increase in these parameters was not reversed by treatment with dexamethasone (Figs. 10, 11 and 12), characterizing the glucocorticoid resistance reported in severe asthma, confirming once again the implantation of the model.

The exudate was evaluated by typical cell morphology. Eosinophils are polymorphonuclear cells that present cytoplasm with dye affinity for eosin, which differs from other PMNs. Macrophages, on the other hand, are mononuclear with a large cytoplasm and have a kidney-shaped nucleus^41,42^.

This results may have been enhanced by the use of a high glycemic index diet, once the literature reports that high glucose levels, can activate signaling pathways responsible for smooth muscle remodeling, as the activation of PKC stimulates the Ras/Raf-1/MEK-1/ERK1/2 pathways, which activate cyclin D1 to increase smooth muscle cell proliferation, causing a thickening of the smooth muscle layer in chronic DM. Additionally, there were increased numbers of myofibroblasts with collagen deposition surrounding the small bronchiole in the chronic diabetes^43^.

Interestingly, the obese group also showed an increase in the inflammatory area, in the thickness of the smooth muscles and in the filling by extracellular matrix, characterizing the inflammatory condition that obesity alone is able to generate. This data corroborates data from the literature that cite that obesity is characterized as a low-grade systemic inflammation, it can affect cellular and molecular processes of the immune system, probably, it also modulates airway inflammation^44^.

As for the treatment with dexamethasone, all the results that showed no reduction in the effect caused by the induction of asthma exacerbated by obesity were expected, being another indication that the methodology was implemented, since in these cases resistance or lesser efficacy of the treatment is cited with corticosteroids.

In view of these results, the implementation of an obesity-exacerbated asthma model is confirmed, characterized by functional and morphologic respiratory changes, becoming a valid methodology for a better understanding of this association, as well as a search for new therapeutic strategies for the treatment of this condition.

## Materials and methods

### Animals

In this research, we used male Wistar rats (*Rattus norvegicus*), 50 days of age, weighing between 220–270 g, obtained from the animal production unit (UPA) of Instituto de Pesquisa em Fármacos e Medicamentos (IPeFarM) of Universidade Federal da Paraíba, maintained under temperature control conditions (22 ± 1 °C) and a 12-h light–dark cycle, with free access to food and water.

#### Ethical statement

The experimental procedures were performed following the principles of guidelines for the ethical use of animals in applied etiology studies^45^ and from the Brazilian Guide for the Production, Maintenance or Use of Animals in Teaching or Scientific Research Activities, from Conselho Nacional de Controle de Experimentação Animal (CONCEA)^46^. Experimental procedures were approved by the Ethics Committee on Animal Use of UFPB (Universidade Federal da Paraíba) (protocol no. 1162100918). In addition, we confirm that all methods are reported in accordance with ARRIVE guidelines^47^.

### Chemicals

Sodium chloride (NaCl), potassium chloride (KCl), magnesium sulfate (MgSO_4_), potassium phosphate (KH_2_PO_4_), calcium chloride (CaCl_2_), glucose, sodium bicarbonate (NaHCO_3_), monobasic sodium phosphate (NaH_2_PO_4_) and dibasic sodium phosphate NaH_2_PO_4_ were obtained from Êxodo Científica (Sumaré, São Paulo, Brazil). Carbamylcholine hydrochloride (CCh), aluminum hydroxide [Al(OH)_3_], ovalbumin (OVA) (grade II and V), nifedipine, aminophylline and dibasic sodium phosphate (Na_2_HPO_4_) were obtained from Sigma-Aldrich (São Paulo, São Paulo, Brazil). Arachidonic acid (AA) and isoprenaline were purchased from Cayman Chemical (Ann Arbor, Michigan, USA). Dexamethasone disodium phosphate injection was purchased from Aché Laboratórios Farmacêuticos (Guarulhos, São Paulo, Brazil).

Absolute alcohol was purchased from Neon (Suzano, São Paulo, Brazil), and xylol and paraffin were purchased from Dinâmica Química Contemporânea LTDA (Indaiatuba, São Paulo, Brazil). Formaldehyde, hematoxylin, eosin and Masson's trichrome were obtained from different brands.

### Experimental groups

The animals were randomly divided into 5 experimental groups, with 8 animals each: control (Ctrl)—fed a standard diet and not sensitized; obese (Ob)—fed a high glycemic index pellet diet (HGLI) and not sensitized; asthmatic (Asth)—fed a standard diet and sensitized; obese asthma (Ob + Asth)—fed HGLI and sensitized; and obese asthma dexamethasone (Ob + Asth + Dexa)—fed HGLI, sensitized and treated with dexamethasone.

### Diets

The animals from the Ctrl and Asth received a standard diet (Nuvilab), and those from the Ob, Ob + Asth and Ob + Asth + Dexa received an HGLI composed of a standard diet (Nuvilab), refined sugar (Alegre) and condensed milk (Camponesa). This diet was characterized as having a high glycemic index, with a value of 77.6, and a high glycemic load, with values of 38.8^7,48^.

#### Diet centesimal composition

The centesimal composition of the standard diet (Nuvilab) and HGLI were determined by moisture analysis at 102 °C, fixed mineral residue obtained after carbonization and incineration in a muffle furnace at 550 °C, proteins by the Kjeldahl method, lipids by the Soxhlet method, while the carbohydrate content was obtained using the high-performance liquid chromatography technique^49^.

### Sensitization and challenges with ovalbumin for asthma induction and dexamethasone treatment

**Figure 13:**
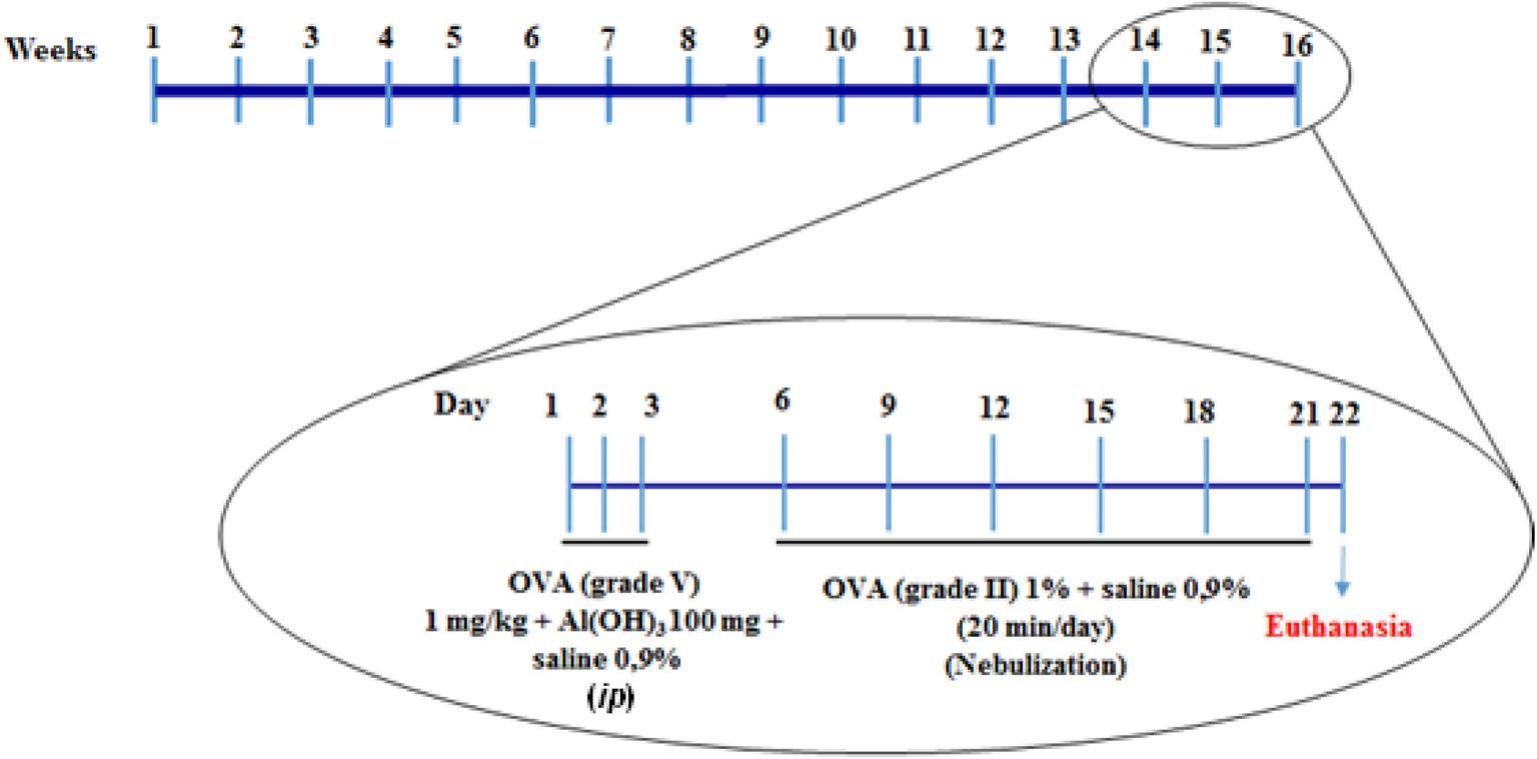
Flowchart and timeline of the study design for asthma. *OVA* ovalbumin, *Al*(*OH*)_*3*_ aluminum hydroxide, *ip* intraperitoneally.

For the induction of asthma in rats, sensitization started at the end of the 13th week after the start of the diets and occurred during the last 22 days (Fig. 1). The protocol followed the methodology described by Salmon et al*.* and Galvão et al*.*^50,51^. Animals in the Ob + Asth + Dexa group during the last 5 days of disease induction received 5 mg/kg per day of dexamethasone intraperitoneally, while the other groups (Ctrl, Ob, Asth and Ob + Asth) received saline via the same route^52^ (Fig. 13).

### Obtaining tracheal rings

The rats were euthanized by anesthetization with ketamine (100 mg/kg) and xylazine (10 mg/kg) *ip*, followed by exsanguination. The trachea was divided into rings, and isometric contractions were evaluated. The integrity of the epithelium was verified, and only rings with intact epithelium were used^53^.

### Nutritional solutions

For the tracheal reactivity protocols, a Krebs nutrient solution was used in an organ bath, adjusted to pH 7.4 (with a solution of HCl or NaOH, 1 N), aerated with a carbogen mixture (95% O_2_ and 5% CO_2_) and maintained at 37 °C. It had the following composition in mM: NaCl (118.0), KCl (4.5), MgSO_4_ (5.7), KH_2_PO_4_ (1.1), CaCl_2_ (2.5), glucose (11.0) and NaHCO_3_ (25.0).

The samples for histological procedures were kept in a buffered formaldehyde solution (10%) with the following composition: formaldehyde (1:10 v/v), NaH_2_PO_4_ (0.04:10 m/v), Na_2_HPO_4_ (0.005:10 m/v) and H_2_O (9:10 v/v).

### Evaluation of experimental obesity

#### Animal weight evolution, food intake and fasting blood glucose

The body mass (g) of the rats was recorded weekly, always on the same days, with the mass gain being calculated by the difference between final and initial body mass.

Food consumption was also calculated weekly, always on the same days, represented by the difference between the food offered and the residual^54^.

After fasted for 8 h, on the first day and on the day of euthanasia, the animals through blood collection from a small incision at the end of the animals' tail, blood glucose was determined using a glucometer (Accu-Chek Performa, Roche Diagnostics, USA)^55^.

#### Murinometric parameters

##### Lee index, body mass index, chest and abdominal circumferences

On the day of euthanasia, the animals were weighed, and the naso-anal length (cm) was used to calculate the Lee index, which is the ratio between the cube root of body mass and the naso-anal length of the animal^56^, and the body mass index (BMI) was characterized by the ratio between the weight and the naso-anal length of the animal squared. The thoracic circumference, located in the posterior portion of the front leg, and the waist circumference, located in the anterior part of the animal's hind leg, were also measured using an anthropometric body measuring tape^28^.

##### Mass of adipose tissue deposits and adiposity index

The inguinal, epididymal and retroperitoneal adipose tissues were weighed, which represent the main components of central adiposity in rats^57^.

The adiposity index was calculated from the sum of the individual masses of the epididymal, inguinal and retroperitoneal fat layers using the formula inguinal fat + epididymal fat + retroperitoneal fat × 100/final body weight^41^.

### Respiratory rate analysis

On days 1, 12 and 21 of the asthma induction protocol, after nebulization (OVA or saline), the animals were submitted to a measurement of tidal volume, respiratory frequency and ventilation using the plethysmography technique in a full body chamber, adapted from the literature^58,59^.

### Evaluation of contractile and relaxant reactivity

#### Evaluation of contractile reactivity to ovalbumin in rat trachea—Schultz–Dale reaction

The tracheal rings were stimulated with 100 µg/mL OVA, and the contraction amplitude was compared between the Ctrl, Ob, Asth, Ob + Asth and Ob + Asth + Dexa^33,60^.

#### Assessment of tracheal contractile reactivity to KCl and CCh in rat trachea

Two cumulative concentration–response curves were induced for the eletromechanical agent KCl^61^ or CCh, a pharmacomechanical agent^62^. Contractile reactivity was evaluated on the basis of the maximum effect (E_max_) and concentration of a substance that produced 50% of its maximal effect (EC_50_) of the contractile agent, calculated from the concentration–response curves obtained, calculated by nonlinear regression^63^.

#### Assessment of tracheal relaxant reactivity to nifedipine, isoprenaline and aminophylline in rat trachea precontracted with CCh

A contraction with 10^–5^ M CCh was induced, and the tonic component was added in cumulative concentrations to nifedipine, a calcium channel blocker^64^, isoprenaline, a β-adrenoceptor agonist receptor^65^ or aminophylline, a nonselective phosphodiesterase inhibitor^40^, until they reached their E_max_. Relaxant reactivity was expressed as the reverse percentage of the initial contraction force elicited by CCh and evaluated on the basis of EC_50_ and E_max_, calculated from concentration–response curves, by nonlinear regression^63^.

### Morphologic evaluations

#### Evaluation of the effects of obesity and asthma on the pulmonary morphology of rats

Samples of lung tissue fragments were collected and fixed in 10% buffered formalin solution. After that, the samples were dehydrated in growing alcohol solutions from 70°GL to absolute, and then the samples were added to a xylol bath. The tissues were embedded in paraffin, cut into a microtome with a thickness of 4 μm, mounted on histological slides, dewaxed in xylol, and hydrated in alcohols in decreasing concentrations. The samples were then treated with Harris' hematoxylin and eosin or with Masson's Trichrome solution. The slides were dehydrated in increasing concentrations of alcohols, diaphanized in xylol, and assembled with Entellan^66^. Quantification was performed using the ImageJ software, using the program's own tools. Five slides were studied per animal and the entire histological region visualized was microphotographed, with an average of 20 photos per animal.

### Statistical analysis

The results were expressed as the percentage of the mean and the standard error of the mean (e.p.m.), and the E_max_ and EC_50_ were compared and analyzed statistically using one-way analysis of variance (ANOVA) followed by the Tukey posttest. The null hypothesis was rejected when p < 0.05. All data were analyzed using GraphPad Prism^®^ version 5.01.

## Supplementary Information

Supplementary Figures.

## Data Availability

The datasets generated and/or analyzed during the current study are not publicly available due to being in a patenting process but are available from the corresponding author on reasonable request.
