# Supplementary material for: Functional and morphologic dysfunctions in the airways of rats submitted to an experimental model of obesity-exacerbated asthma
Source: Sci Rep. 2022 Jun 9;12:9540. doi: 10.1038/s41598-022-13551-0 (PMC9184493; doi:10.1038/s41598-022-13551-0)
Supplement: Supplementary file 1 — Supplementary Figures. [file 41598_2022_13551_MOESM1_ESM.docx]

**Supplementary Information**

**Supplementary figure S1 –** Original records representing respiratory function of animals from Ctrl, Ob, Asth, Ob + Asth and Ob + Asth + Dexa, on days 1, 12 and 21.

**Ctrl**

Day 21

Day 12

Day 1


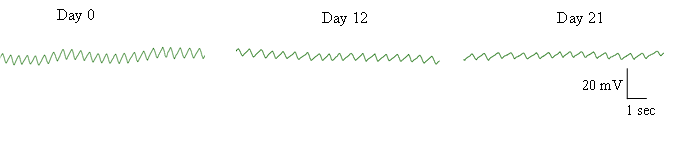


**
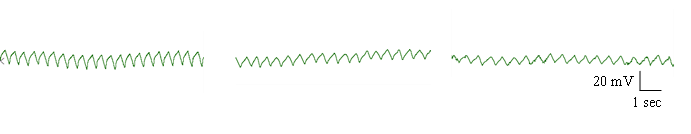
Ob**

**Asth**

**
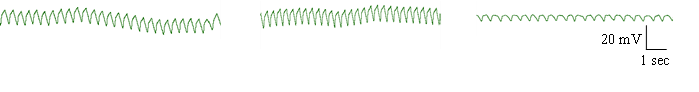
**

**
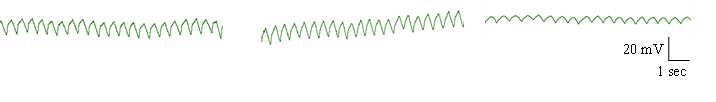
Ob + Asth**

**Ob + Asth + Dexa**

**
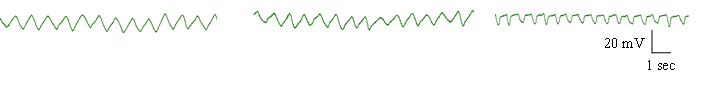
**

**Supplementary figure S2 –** Original records representative of tracheal reactivity induced with 100 µg/mL of OVA in rats from Ctrl, Ob, Asth, Ob + Asth and Ob + Asth + Dexa.

**
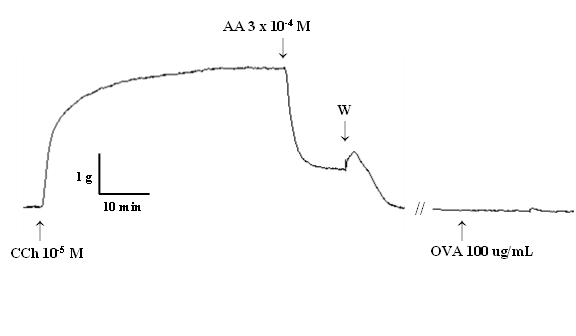

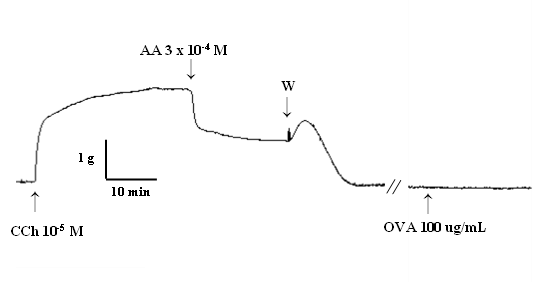
**

**Ctrl Ob**


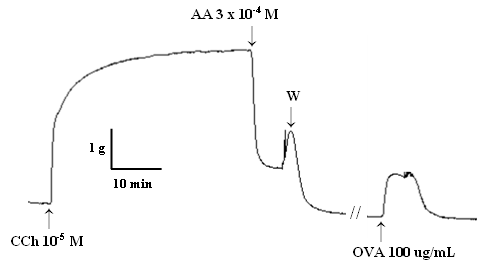
**Asth Ob + Asth**

**
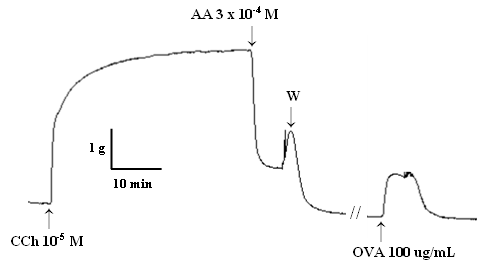
**

**Ob + Asth + Dexa**


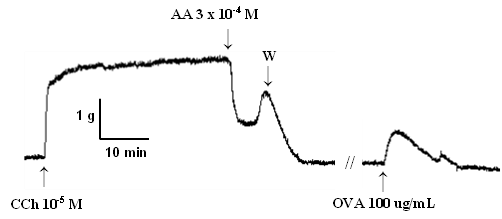


CCh: carbachol; AA: arachidonic acid; W: washing; OVA: ovalbumin.

**Supplementary figure S3 –** Original records representative of contractile reactivity to potassium Chloride (KCl) in the trachea of animals from Ctrl, Ob, Asth, and Ob + Asth.


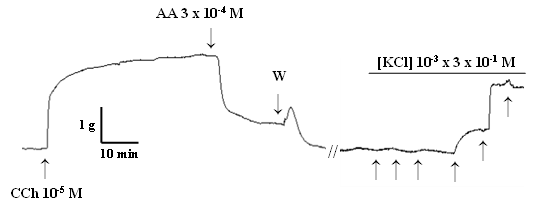


**[KCl] 10^-3^ – 3 x 10^-1^M**

**Ctrl**


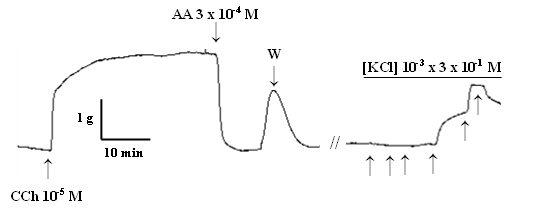


**[KCl] 10^-3^ – 3 x 10^-1^M**

**Ob**

**Asth**


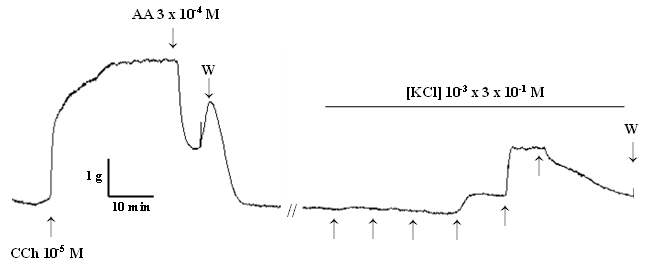


**[KCl] 10^-3^ – 3 x 10^-1^M**

**Ob + Asth**


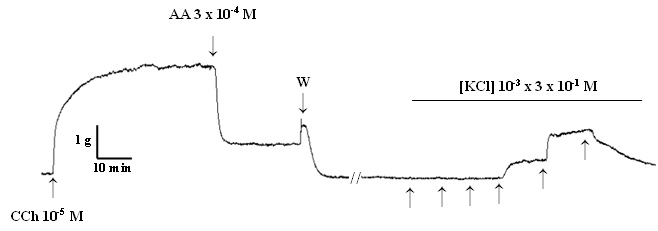


**[KCl] 10^-3^ – 3 x 10^-1^M**

CCh: carbachol; AA: arachidonic acid; W: washing; KCl: potassium chloride.

**Supplementary figure S4 –** Original records representative of contractile reactivity to CCh in the trachea of animals from Ctrl, Ob, Asth, Ob + Asth and Ob + Asth + Dexa.


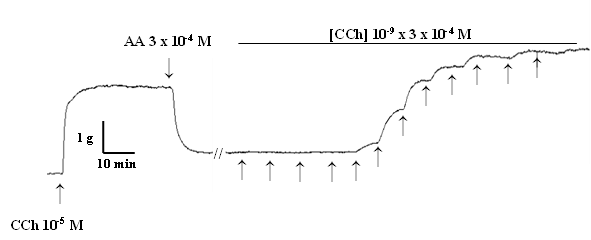


**[CCh] 10^-9^ – 3 x 10^-4^ M**

**Ctrl**

**Ob**


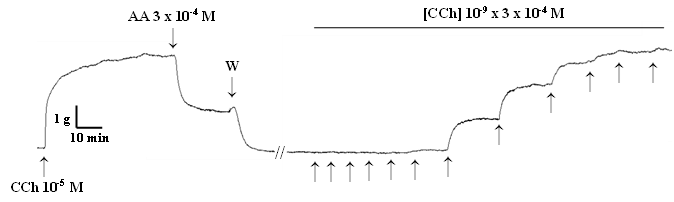


**[CCh] 10^-9^ – 3 x 10^-4^ M**

**Asth**


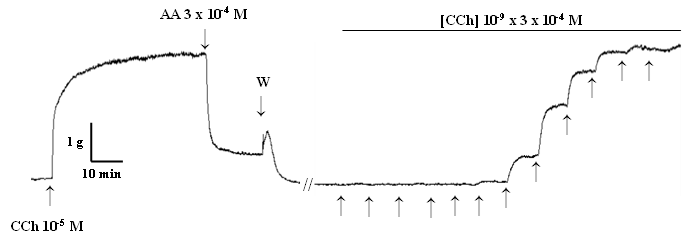


**[CCh] 10^-9^ – 3 x 10^-4^ M**


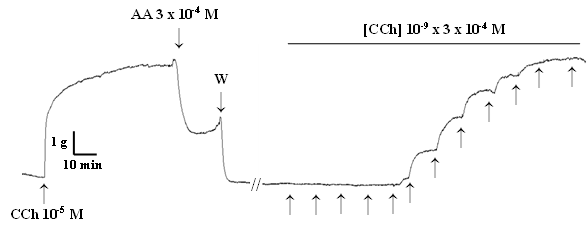


**[CCh] 10^-9^ – 3 x 10^-4^ M**

**Ob + Asth**


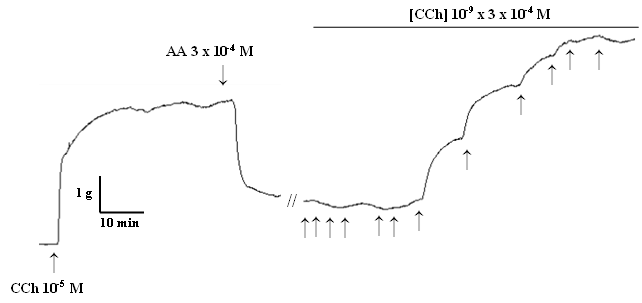


**[CCh] 10^-9^ – 3 x 10^-4^ M**

**Ob + Asth + Dexa**

CCh: carbachol; AA: arachidonic acid; W: washing.

**Supplementary figure S5 –** Original records representative of relaxing reactivity to nifedipine from the trachea of animals from Ctrl, Ob, Asth and Ob + Asth.

**Ctrl**


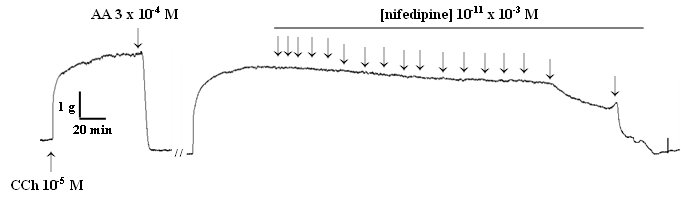


**[nifedipine] 10^-11^ – 10^-3^ M**

**Ob**


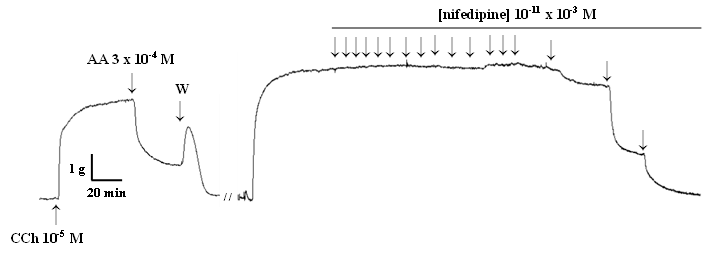


**[nifedipine] 10^-11^ – 10^-3^ M**

**Asth**


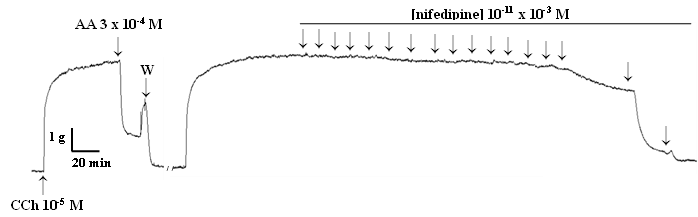


**[nifedipine] 10^-11^ – 10^-3^ M**

**Ob + Asth**


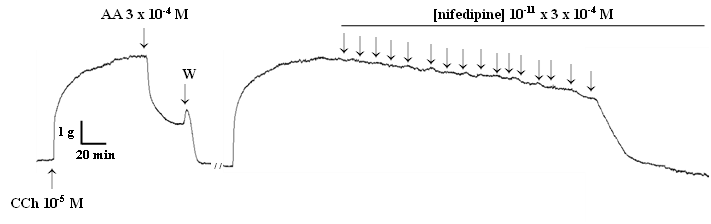


**[nifedipine] 10^-11^ – 3 x 10^-4^ M**

CCh: carbachol; AA: arachidonic acid; W: washing.

**Supplementary figure S6–** Original records representative of relaxing reactivity to isoprenaline from the trachea of animals from Ctrl, Ob, Asth and Ob + Asth.

**Ctrl**


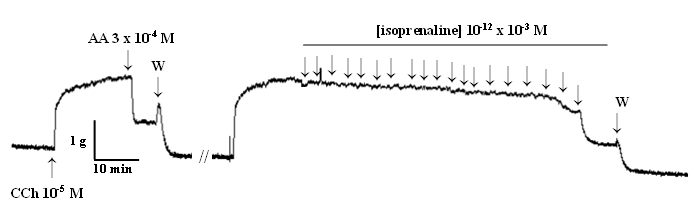


**[isoprenaline] 10^-12^ – 10^-3^ M**

**Ob**


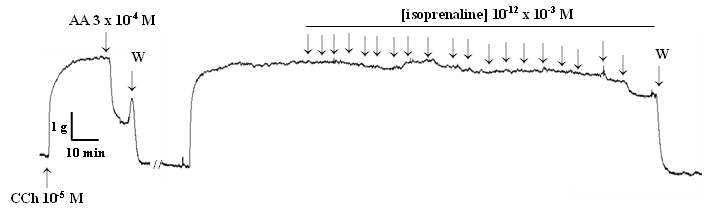


**[isoprenaline] 10^-12^ – 10^-3^ M**

**Asth**


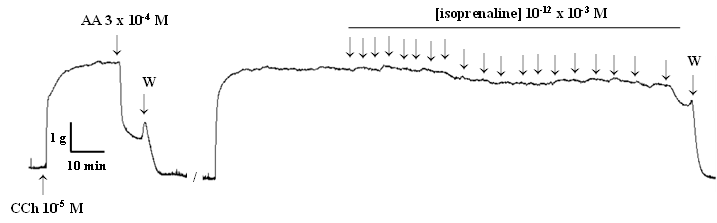


**[isoprenaline] 10^-12^ – 10^-3^ M**

**
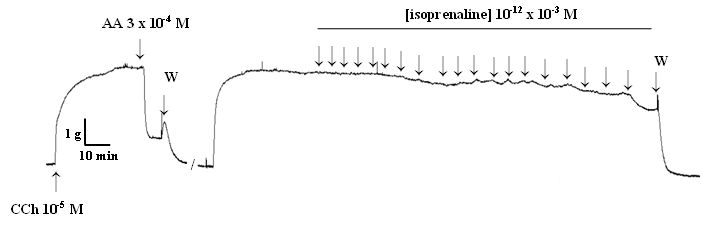
Ob + Asth**

CCh: carbachol; AA: arachidonic acid; W: washing.

**Supplementary figure S7–** Original records representative of relaxing reactivity to aminophylline from the trachea of animals from Ctrl, Ob, Asth and Ob + Asth.

**Ctrl**


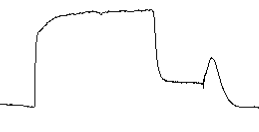

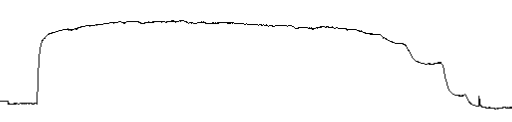


//

**↑**

**↑**

**CCh 10^-5^ M**

**AA 3 x 10^-4^ M**

**[**aminophylline**] 10^-9^ - 10^-3^ M**

**1 g**

**10 min**

**↑**

**↑**

**↑**

**↑**

**↑**

**↑**

**↑**

**↑**

**↑**

**↑**

**↑**

**↑**

**↑**

**↑**

**↑**

**W**


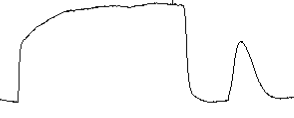

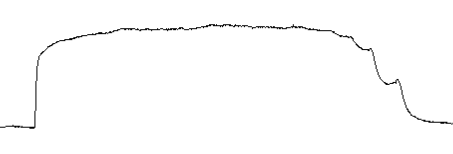


//

**↑**

**↑**

**CCh 10^-5^ M**

**AA 3 x 10^-4^ M**

**[**aminophylline**] 10^-9^ - 10^-3^ M**

**1 g**

**10 min**

**↑**

**↑**

**↑**

**↑**

**↑**

**↑**

**↑**

**↑**

**↑**

**↑**

**↑**

**↑**

**↑**

**↑**

**↑**

**W**

**Ob**

**Asth**


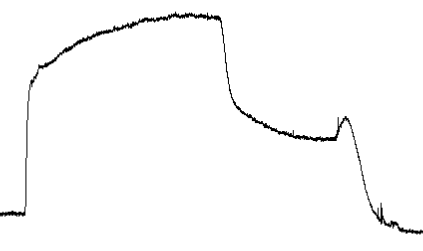

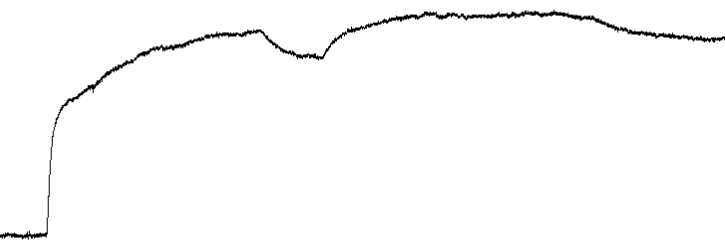

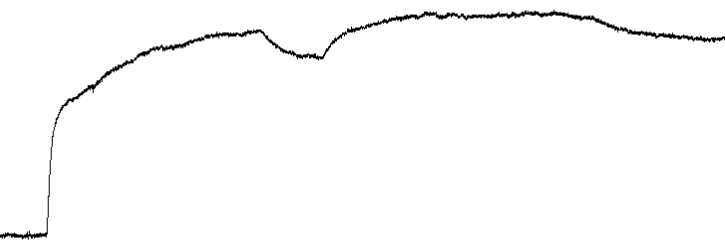

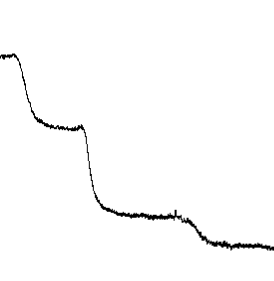


//

**↑**

**↑**

**CCh 10^-5^ M**

**AA 3 x 10^-4^ M**

**[**aminophylline**] 10^-6^ - 3 x 10^-3^ M**

**1 g**

**10 min**

**↑**

**↑**

**↑**

**↑**

**↑**

**↑**

**↑**

**↑**

**↑**

**W**

**Ob + Asth**


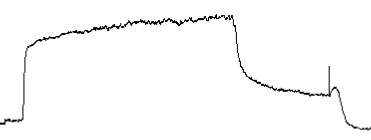

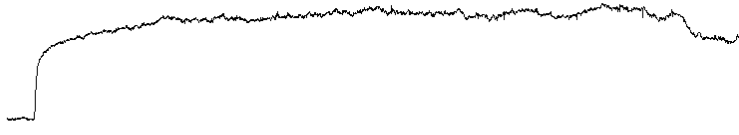

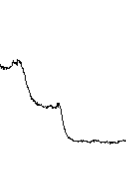


//

**↑**

**↑**

**CCh 10^-5^ M**

**AA 3 x 10^-4^ M**

**[**aminophylline**] 10^-9^ - 10^-3^ M**

**1 g**

**10 min**

**↑**

**↑**

**↑**

**↑**

**↑**

**↑**

**↑**

**↑**

**↑**

**↑**

**↑**

**↑**

**↑**

**↑**

**↑**

**W**

CCh: carbachol; AA: arachidonic acid; W: washing.
